# The PRECIsE study: a prospective, multicenter study of shape-sensing robotic-assisted bronchoscopy with 2 years of follow-up

**DOI:** 10.1093/annalsats/aaoag049

**Published:** 2026-03-03

**Authors:** David E Ost, Erik E Folch, Janani S Reisenauer, Adnan Majid, Roberto F Casal, Colleen Keyes, Mihir S Parikh, Javier Diaz-Mendoza, Sebastian Fernandez-Bussy, Michael J Simoff

**Affiliations:** Department of Pulmonary Medicine, The University of Texas MD Anderson Cancer Center, Houston, TX, United States; Department of Pulmonary and Critical Care Medicine, Massachusetts General Hospital–Harvard Medical School, Boston, MA, United States; Department of Pulmonary Medicine and Thoracic Surgery, Mayo Clinic, Rochester, MN, United States; Department of Thoracic Surgery and Interventional Pulmonology, Beth Israel Deaconess Medical Center–Harvard Medical School, Boston, MA, United States; Department of Pulmonary Medicine, The University of Texas MD Anderson Cancer Center, Houston, TX, United States; Department of Pulmonary and Critical Care Medicine, Massachusetts General Hospital–Harvard Medical School, Boston, MA, United States; Department of Thoracic Surgery and Interventional Pulmonology, Beth Israel Deaconess Medical Center–Harvard Medical School, Boston, MA, United States; Department of Pulmonary and Critical Care Medicine, Henry Ford Hospital, Detroit, MI, United States; Department of Pulmonary Medicine, Mayo Clinic, Jacksonville, FL, United States; Department of Pulmonary and Critical Care Medicine, Henry Ford Hospital, Detroit, MI, United States

**Keywords:** peripheral pulmonary nodules, bronchoscopy, sensitivity for malignancy, diagnostic yield, lung cancer

## Abstract

**Rationale:**

Guidelines recommend nonsurgical biopsy for indeterminate pulmonary lesions >8 mm. Shape-sensing robotic-assisted bronchoscopy (ssRAB) is growing in use to biopsy small pulmonary nodules; however, current literature is limited to single-center studies with limited follow-up.

**Objectives:**

This study, PRECIsE, assessed safety and performance of the first ssRAB iteration across multiple sites and nascent users.

**Methods:**

This was a prospective, multicenter, observational study evaluating ssRAB without cone-beam computed tomography guidance in patients with nodules 10 to 30 mm located in or beyond the sub-segmental airways. Patients were followed for 2 years; the primary endpoint was sensitivity for malignancy with diagnostic yield, and safety as secondary endpoints. Multilevel logistic regression models were used to control for factors associated with sensitivity and diagnostic yield.

**Results:**

A total of 305 procedures were performed across 6 centers. Median nodule size was 17.0 mm (IQR, 14.0-23.0) with bronchus sign present in 37% of cases. Sensitivity for malignancy through 2 years was 81.3% (95% CI, 75.7%-86.1%). Multilevel modeling demonstrated that female sex, smaller nodule size, lower lobe location, semi-solid density, and higher body mass index were associated with lower sensitivity. Diagnostic yield was 74.1% (95% CI, 68.8%-78.9%) according to the American Thoracic Society/American College of Chest Physicians (ATS/ACCP) criteria and 77.5% (95% CI, 72.4%-81.8%) according to the intermediate criteria. Multilevel modeling demonstrated a nonsignificant site/center-level effect on ATS/ACCP diagnostic yield (*P* = .36). Pneumothorax requiring intervention was 1.6% (5/305); bleeding was 1.0% (3/305) with 2 Nashville grade 2 events and one Nashville grade 3 event.

**Conclusions:**

The first iteration of ssRAB demonstrated encouraging performance and a strong safety profile among nascent users for the biopsy of small peripheral nodules.

## Background

Guidelines recommend nonsurgical biopsy for indeterminate pulmonary lesions larger than 8 mm, using either transthoracic needle aspiration (TTNA) or bronchoscopy.^1^^,^^2^ The optimal diagnostic approach depends on factors such as lesion size, location, presence of adenopathy, and the need for mediastinal staging. TTNA is often preferred for solitary pulmonary nodules due to its high sensitivity for malignancy (≥90%)^1^ and cost-effectiveness compared with bronchoscopy.^3^ However, TTNA sensitivity may be lower in smaller nodules^4^^,^^5^ and it has a higher risk of pneumothorax (25.9%).^6^ In contrast, bronchoscopy is preferred for patients with peripheral lung lesions and concurrent hilar or mediastinal lymphadenopathy, as it enables simultaneous mediastinal staging.^3^^,^^7^ Consequently, bronchoscopy-first strategies with mediastinal sampling result in fewer tests, lower costs, reduced complications, and fewer futile surgeries compared with TTNA-first approaches in such patients.^8^

Before the advent of robotic bronchoscopy, guided bronchoscopy techniques yielded lower diagnostic sensitivity for malignancy, particularly for nodules <20 mm. A recent meta-analysis reported a pooled sensitivity of 72% for bronchoscopy with radial endobronchial ultrasound (rEBUS), while a randomized trial comparing standard and ultrathin bronchoscopy with rEBUS reported a sensitivity of 57.8%.^9^^,^^10^ Electromagnetic navigation (EMN) bronchoscopy improved sensitivity to 68.8% to 77%, but these values remain lower than those reported for TTNA, highlighting the limitations of these approaches.^11^^,^^12^

Shape-sensing robotic-assisted bronchoscopy (ssRAB) (Ion Endoluminal System, Intuitive Surgical, Sunnyvale, CA, United States), employs a multicore shape-sensing fiber within a flexible 3.5-mm outer diameter catheter. This fiber measures catheter position >300 times per second every 40 microns along its length, ensuring precise navigation. The catheter tip maintains stability during biopsy or other procedures when not actively guided.^13^^,^^14^

Existing literature on ssRAB is largely limited to single-center, retrospective studies or those with short follow-up periods. The PRECIsE study (NCT03893539), the first multicenter, prospective study with concurrent enrollment and 2-year follow-up, aims to evaluate the performance of the first commercial iteration of ssRAB. The primary objective is to quantify diagnostic sensitivity for malignancy, diagnostic yield, and complication rates across multiple sites and robotic-naive users. A secondary objective is to identify factors associated with these outcomes.

## Methods

The PRECIsE study is a prospective, multicenter, observational cohort study involving proceduralists new to robotic-assisted bronchoscopy (RAB). The study was approved by each institution’s review board (see online supplement), and informed consent was obtained for all patients. All investigators underwent standardized training on the ssRAB system. A lead-in phase allowed each investigator to perform 5 to 10 procedures with 30-day follow-up to gain familiarity with the system; these cases were excluded from the study. The study enrolled consecutive adult patients with pulmonary nodules 10 to 30 mm in diameter, located at or beyond the subsegmental airways. Enrollment occurred in 2 stages: the clinical utility stage (stage 1) included the first 25 cases per center, followed by the early performance stage (stage 2), which included the subsequent 25 to 30 cases per center. Complete study eligibility criteria are provided in the online supplement.

### Study procedure

**Figure 1:**
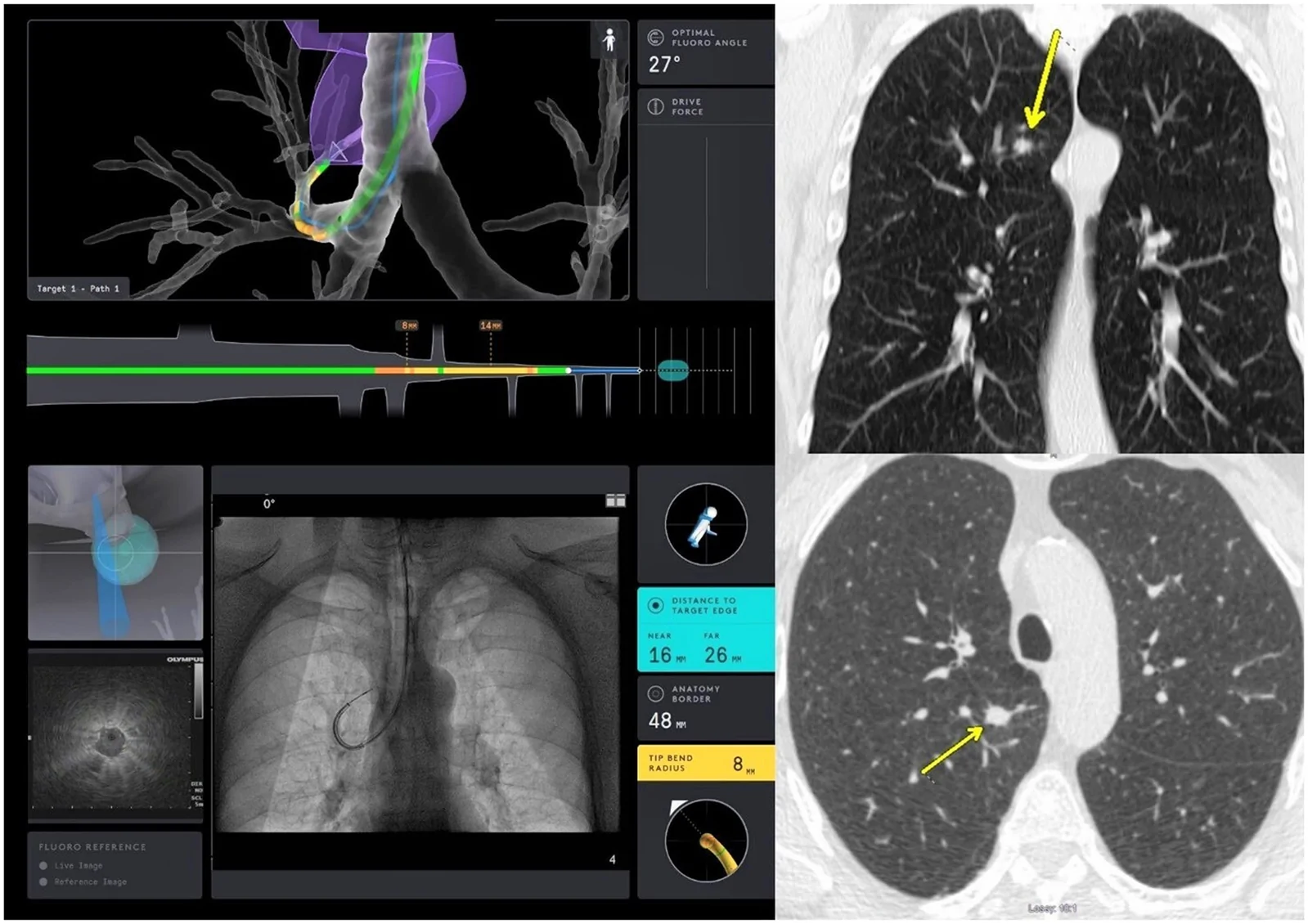
Example of shape-sensing robotic-assisted bronchoscopy of a right upper lobe lung nodule (arrow) with concurrent radial EBUS imaging.

Preprocedure navigational planning was performed using PlanPoint software (Intuitive Surgical), which facilitated pathway generation and integration of anatomic boundaries. Biopsies were conducted under general anesthesia with paralytics and mechanical ventilation. No specific ventilation strategies were mandated. Following an airway survey, the ssRAB system was docked, and registration was performed to align the segmented airway model with the patient’s anatomy. The catheter was navigated to the target nodule under real-time visualization and stabilized for biopsy after removal of the vision probe. Two-dimensional fluoroscopy was used in all cases, supplemented by rEBUS at all centers (Figure 1). Biopsy tools included proprietary 19G, 21G, or 23G biopsy needles (Flexision, Intuitive Surgical), biopsy forceps, cytology brushes, and bronchoalveolar lavage; cryobiopsy was not performed. Although tool sequence was not standardized, use of the biopsy needle as the initial tool with sampling from multiple nodule quadrants was recommended. Rapid on-site evaluation (ROSE) was available at 5 centers and used according to proceduralist practice. Three-dimensional imaging, such as cone-beam computed tomography (CBCT), was prohibited for study biopsies; biopsies involving CBCT contributed to the reference standard but were excluded from performance outcome calculations. Pneumothorax was assessed by fluoroscopy at procedure completion and by chest radiography at least 1 hour postprocedure. If symptoms occurred after the chest radiograph and a late pneumothorax was detected or if a pneumothorax was reported at a follow-up visit through 30 days, it was included in the reported rate. Lymph node staging via linear endobronchial ultrasound-guided transbronchial needle aspiration (EBUS-TBNA) was performed after biopsy per institutional protocols.

### Outcomes

The primary endpoint was sensitivity for malignancy at 1 and 2 years. Secondary outcomes included procedure characteristics, diagnostic yield, and rates of pneumothorax and airway bleeding. The reference standard for malignancy was defined as follows: Patients with an index biopsy positive for malignancy were classified as true positives (TP). For patients with nonmalignant index biopsies, the underlying diagnosis was determined through one of several pathways. A specific benign diagnosis (eg, hamartoma) on index biopsy was accepted as the true state. Other specific benign diagnoses (eg, histoplasmosis) were confirmed through follow-up (see online supplement for details). For nonspecific index biopsies, the diagnosis was established via follow-up biopsy or 2-year computed tomography (CT) surveillance. Patients with nonmalignant index biopsies later diagnosed with cancer through subsequent sampling, surgery, or oncologic treatment initiation were classified as false negatives (FN). Similarly, if the index biopsy of a peripheral nodule was nonmalignant but malignancy was identified via CBCT-aided ssRAB during the same procedure or if EBUS-TBNA revealed malignant lymph nodes, the ssRAB result was considered FN. True negatives (TN) were patients with a specific nonmalignant diagnosis (eg, hamartoma) confirmed by subsequent intervention or 2-year radiographic surveillance showing no nodule growth. Sensitivity for malignancy was calculated as TP / (TP + FN). The probability of malignancy in nondiagnostic cases was determined as the proportion of patients with nondiagnostic index biopsies later proven malignant. If patients with nondiagnostic results were lost to follow-up prior to 2 years, they were considered as TN for the primary analysis. However, we performed sensitivity analysis, in which all patients lost to follow-up were counted as FN and recalculated sensitivity.

Diagnostic yield was initially defined as the number of diagnostic cases divided by the total cases attempted. A result was considered diagnostic if the pathology showed malignancy, a specific benign diagnosis (eg, hamartoma, fungal infection), or a nonspecific nonmalignant finding (eg, inflammation) adjudicated as benign by principal investigators. Results indicating suspicion for malignancy, atypia, lung parenchyma, or benign bronchial epithelial cells were classified as nondiagnostic. Unique nonspecific findings deemed diagnostic were reviewed with the investigator or adjudicated with other site investigators. For this manuscript, intermediate diagnostic yield refers to this original study definition. Following the study’s initiation, the American Thoracic Society/American College of Chest Physicians (ATS/ACCP) published stricter recommendations for diagnostic yield reporting.^15^ Diagnostic yield was recalculated using the ATS/ACCP definition for analysis, with both definitions detailed in the contingency tables (Table S1).

Primary safety outcomes were pneumothorax and airway bleeding, graded using the Nashville scale.^16^ Pathology reports were verified for all enrolled subjects.

### Statistical analysis

Continuous variables are reported as median with interquartile range (IQR). Categorical variables are described as counts and proportions of the totals; for subgroup comparisons, a χ^2^ or Fisher exact test was used with *P* value <.05 considered statistically significant. Statistical software SAS version 9.4 (SAS Institute, Cary, NC, United States) was used.

Multilevel logistic regression models, where center was modeled as a random effect, investigated the association of preprocedure variables on diagnostic yield and sensitivity for malignancy. Baseline parameters included demographics, presence of comorbidities, and nodule characteristics; procedure variables such as number of needle passes were not included due to confounding from variables such as rEBUS visualization and ROSE results. Univariate logistic regression was performed as a first step, and variables with a *P* value ≤.2 were selected as candidates for model selection using Akaike information criterion–based stepwise regression. The selected variables from the stepwise regression were used as covariates in a multilevel logistic regression model. For diagnostic yield, the multilevel model included center and physician as nested random effects, with physician nested within centers. For sensitivity of malignancy, due to a reduced sample size, the multilevel model included center as a random effect. For patients with multiple nodules in the diagnostic yield model, each nodule was treated as an independent observation. Correlations among nodules from the same patient were not modeled. Sensitivity of malignancy was analyzed at the patient level. We performed a post hoc analysis, in which only the first nodule biopsied was counted and a separate analysis of nodule size stratified by quartile.

## Results

A total of 365 patients were enrolled across 6 centers from March 29, 2019 to May 26, 2021. Notably the COVID-19 pandemic impacted enrollment due to restrictions for procedures or research activity. Among the 365 subjects, 60 were enrolled in the lead-in stage, 150 in the clinical utility stage, and 155 in the early performance stage. Results from the lead-in phase have been reported^13^ and are not included.

Subject demographics and nodule characteristics are presented in Table 1. Median nodule size was 17.0 mm (IQR, 14.0-23.0). Nodules were located at a median seventh (IQR, 6-8) generation with a median distance to pleura of 11.0 mm (IQR, 1.0-23.0). Bronchus sign was present in 37.4% of cases.

**Table 1:** Demographics, clinical history, and nodule characteristics.

Variable	**Stage 1 (*n*** = **150)**	**Stage 2 (*n*** = **155)**	**Combined (*N*** = **305)**
**Age, y, median, (IQR)**	68.9 (62.3-74.8)	69.3 (64.0-75.9)	69.0 (63.2-75.5)
**BMI, kg/m^2^, median (IQR)**	26.7 (23.1-30.8)	27.4 (22.7-31.6)	27.1 (23.0-31.0)
**Sex, No. (%)**			
** Female**	88 (58.7)	89 (57.4)	177 (58.0)
** Male**	62 (41.3)	66 (42.6)	128 (42.0)
**Preprocedure risk of malignancy** ^a^ **, %, median (IQR)**	45.2 (27.8-65.0)	36.6 (21.7-56.8)	41.4 (24.5-64.4)
**Smoking history, No. (%)**			
** Current**	38 (25.3)	40 (25.8)	78 (25.6)
** Never**	78 (52.0)	79 (51.0)	70 (23.0)
** Previous**	34 (22.7)	36 (23.2)	157 (51.5)
**Pulmonary comorbidities, No. (%)**			
** Pulmonary hypertension**	5 (3.3)	1 (0.6)	6 (2.0)
** COPD**	55 (36.7)	59 (38.1)	114 (37.4)
** Emphysema**	50 (33.3)	60 (38.7)	110 (36.1)
** Bronchitis/chronic bronchitis**	13 (8.7)	7 (4.5)	20 (6.6)
** Dyspnea/shortness of breath**	45 (30.0)	44 (28.4)	89 (29.2)
** Asthma**	13 (8.7)	19 (12.3)	32 (10.5)
** Bronchiectasis**	10 (6.7)	6 (3.9)	16 (5.2)
** Hemoptysis**	0 (0.0)	0 (0.0)	0 (0.0)
** Pulmonary fibrosis**	3 (2.0)	1 (0.6)	4 (1.3)
**Nodule size—largest diameter, mm, median (IQR)**	18.0 (14.6-24.0)	16.0 (13.0-22.0)	17.0 (14.0-23.0)
**Lobe location, No. (%)**			
** RUL**	55 (33.7)	57 (33.1)	112 (33.4)
** RML**	19 (11.7)	13 (7.6)	32 (9.6)
** RLL**	24 (14.7)	30 (17.4)	54 (16.1)
** LUL**	42 (25.8)	48 (27.9)	90 (26.9)
** LLL**	23 (14.1)	24 (14.0)	47 (14.0)
**Closest distance from pleura, mm, median (IQR)**	12.0 (2.0-24.0)	10.0 (0.0-21.0)	11.0 (1.0-23.0)
**Bronchial generation count, median (IQR)**	7.0 (6.0-8.0)	7.0 (6.0-8.0)	7.0 (6.0-8.0)
**CT bronchus sign, No. (%)**			
** Present**	66 (44.0)	48 (31.0)	114 (37.4)
** Absent**	83 (55.3)	107 (69.0)	190 (62.3)
**Nodule type, No. (%)**			
** Solid**	131 (80.4)	152 (88.4)	283 (84.5)
** Semisolid**	30 (18.4)	20 (11.6)	50 (14.9)

Abbreviations: BMI, body mass index; COPD, chronic obstructive pulmonary disease; CT, computed tomography; IQR, interquartile range; LLL, left lower lobe; LUL, left upper lobe; RLL, right lower lobe; RML, right middle lobe; RUL, right upper lobe.

a Swensen model.

Descriptive procedure characteristics are reported in Table 2. Median procedure time was 56.5 minutes (IQR, 41.0-79.5). rEBUS visualization was achieved in 89.2% of cases with a concentric view obtained in 56.6% of visualized cases. Of the concentric cases, approximately 17% had an initial eccentric view with a concentric view achieved through catheter manipulation or tool passes that facilitated better rEBUS positioning. The majority of subjects (228/305 [74.75%]) had staging performed during the same procedure as the peripheral nodule biopsy. While staging was performed after the nodule biopsy was complete, 208/228 (91.2%) of subjects staged had no evidence of nodal disease identified through EBUS-TBNA staging. A total of 20/228 (8.8%) subjects had nodal disease identified through concurrent EBUS-TBNA: 9/228 (3.95%) N1, 10/228 (4.4%) N2, and 1/228 (0.4%) N3.

**Table 2:** Procedure characteristics and safety outcomes.

Variable	**Stage 1 (*n*** = **150)**	**Stage 2 (*n*** = **155)**	**Combined (*N*** = **305)**
**Duration, min, median (IQR)**			
** Procedure time** ^a^	57.0 (42.0-79.0)	55.0 (39.0-80.0)	56.5 (41.0-79.5)
** Navigation time**			
** From procedure start**	12.0 (9.0-18.5)	11.0 (7.0-18.0)	12.0 (8.0-18.0)
** From registration end**	5.0 (3.0-9.0)	6.0 (3.0-11.0)	5.0 (3.0-11.0)
** First rEBUS visualization** ^b^	14.0 (10.0-20.0)	11.0 (8.0-17.0)	13.0 (9.0-19.0)
** Fluoroscopy time** ^c^	6.5 (4.4-10.0)	7.3 (4.8-11.0)	7.1 (4.5-10.6)
**Distance from catheter tip to virtual target, mm, median (IQR)**	6.0 (2.0-11.0)	5.0 (1.0-8.0)	5.0 (1.0-10.0)
**EBUS visualization**	140 (93.3)	132 (85.2)	272 (89.2)
** Concentric view**	76 (54.3)	78 (59.1)	154 (56.6)
** Eccentric view**	64 (45.7)	54 (40.9)	118 (43.4)
**Navigation success** ^d^	148 (98.7)	151 (97.4)	299 (98.0)
**Biopsy success** ^e^	141 (94.6)	148 (96.7)	289 (95.7)
**Biopsy workflow**			
** Catheter tip positions, *n*, median (IQR)**	4.0 (2.0-7.0)	4.0 (2.0-8.0)	4.0 (2.0-7.0)
** Biopsy tool passes, *n*, median (IQR)** ^f^	12.0 (8.0-16.0)	14.0 (10.0-18.0)	13.0 (9.0-17.0)
**Cases with Flexision needle**	149 (99.3)	152 (98.1)	301 (98.7)
** 19G**	38 (25.3)	39 (25.2)	77 (25.2)
** 21G**	102 (68.0)	107 (69.0)	209 (68.5)
** 23G**	47 (31.3)	52 (33.5)	99 (32.5)
**Cases with forceps**	94 (62.7)	111 (71.6)	205 (67.2)
**Cases with cytology brush**	61 (40.7)	68 (43.9)	129 (42.3)
**Tool sequence** ^g^			
** Needle**	78 (43.1)	55 (28.4)	133 (35.5)
** Needle, forceps**	28 (15.5)	42 (21.6)	70 (18.7)
** Needle, forceps, brush**	23 (12.7)	33 (17.0)	56 (14.9)
** Needle, brush, forceps**	19 (10.5)	11 (5.7)	30 (8.0)
**Concurrent lymph node staging**	120 (80.0)	108 (69.7)	228 (74.7)
**Pneumothorax**	7 (4.7)	7 (4.5)	14 (4.6)
** Requiring intervention**	1 (0.7)	4 (2.6)	5 (1.6)
** Not requiring intervention**	6 (4.0)	3 (1.9)	9 (3.0)
**Airway bleeding**	2 (1.3)	1 (0.7)	3 (1.0)
** Nashville grade 2**	2 (1.3)	0 (0.0)	2 (0.65)
** Nashville grade 3** ^h^	0 (0.0)	1 (0.6)	1 (0.3)
**SAEs** ^i^	1 (0.7)	1 (0.6)	2 (0.65)
** Exacerbation of acute respiratory failure**	1	0	1
** Cardiac arrythmia requiring intervention**	0	1	1
**Non–SAEs** ^j^	4 (2.7)	4 (2.6)	8 (2.6)
** Scant hemoptysis**	1	3	4
** Cough**	1	2	3
** Pulmonary edema**	1	0	1
** Viral illness/upper respiratory infection**	1	0	1
** Hoarseness**	1	0	1
** Sore throat**	1	0	1
** Prolonged (>1 min) hypoxemia**	0	1	1

Data are presented as No. (%) unless otherwise indicated. Safety event rates are reported at the subject level; subjects may have had more than one event.

Abbreviations: EBUS, endobronchial ultrasound; IQR, interquartile range; rEBUS, radial endobronchial ultrasound; SAE, serious adverse event.

a Catheter in to catheter out.

b Procedure start to first rEBUS visualization.

c During Ion procedure only.

d Defined as rate of procedures where the catheter tip reached within 2 cm of the virtual target with a tool trajectory in the direction of the target.

e Defined as rate of procedures where samples were obtained and pathology showed characteristics other than benign bronchial epithelial cells.

f rEBUS not included; includes all tools used (needle, forceps, brushes).

g Top 4 tool sequences presented.

h Event graded 3 due to a brief use of Fogarty balloon for tamponade. Selective intubation was not performed.

i SAEs are not inclusive of pneumothorax requiring intervention or airway bleeding. One patient with congestive heart failure and restrictive lung disease experienced an exacerbation of acute respiratory failure resolved with supplemental oxygen and overnight admission, and one patient with intraprocedure bradycardia resolved with atropine.

j Non-SAEs were Common Terminology Criteria for Adverse Events grade ≤2.

### Safety

Safety results are reported in Table 2. Pneumothorax occurred in 14/305 (4.6% [95% CI, 2.5%-7.6%]) patients, with 5/305 (1.6% [95% CI, 0.5%-3.8%]) requiring intervention. Airway bleeding occurred in 3/305 (1.0% [95% CI, 0.2%-2.3%]) patients, 2 Nashville grade 2 due to wedging or tamponade with the catheter only, and one Nashville grade 3 requiring a Fogarty balloon for tamponade but without selective intubation.

### Performance

Performance outcomes are presented in Table 3. Sensitivity for malignancy using follow-up date through 1 year was 191/229 (83.4% [95% CI, 77.9%-88.0%]). An additional 6 FN were identified during the second year, leading to a sensitivity of 191/235 (81.3% [95% CI, 75.7%-86.1%]). Six subjects were lost to follow-up with no information available despite chart review. In sensitivity analysis, we counted these 6 patients as FN, leading to a minimum sensitivity estimate of 191/241 (79.3% [95% CI, 73.6%-84.2%]).

**Table 3:** Performance outcomes.

Variable	**Stage 1 (*n*** = **150)**	**Stage 2 (*n*** = **155)**	**Combined (*N*** = **305)**	*P* value
**Diagnostic yield—intermediate, No. (%) [95% CI]**	112 (75.2) [67.6-81.4]	122 (79.7) [72.5-85.8]	234 (77.5) [72.4-81.8]	
**Biopsy not attempted, No. (%)**	1 (0.67)	2 (1.3)	3 (0.98)	
**Diagnostic yield, 1-2 cm, No. (%)**	64/90 (71.1)	81/104 (77.9)	145/194 (74.7)	.13
**Diagnostic yield, >2-3 cm, No. (%)**	48/59 (81.4)	41/49 (83.7)	89/108 (82.4)	
**Diagnostic yield, bronchus sign present, No. (%)**	49/66 (74.2)	37/48 (77.1)	86/114 (75.4)	.36
**Diagnostic yield, bronchus sign absent, No. (%)**	62/82 (75.6)	85/105 (81.0)	147/187 (78.6)	
**Flexision needle diagnosis, No. (%)**	102 (89.5)	99 (81.1)	201 (85.2)	
**Diagnostic yield—ATS/ACCP, No. (%) [95% CI]**	109 (72.7) [64.8-79.6]	117 (75.5) [67.9-82.0]	226 (74.1) [68.8-78.9]	
**Diagnostic yield, 1-2 cm, No. (%)**	62/97 (68.1)	77/106 (72.6)	139/197 (72.6)	.06
**Diagnostic yield, >2-3 cm, No. (%)**	47/59 (79.7)	40/49 (81.6)	87/108 (80.6)	
**Diagnostic yield, bronchus sign present, No. (%)**	48/66 (72.7)	36/48 (75.0)	84/114 (73.7)	.92
**Diagnostic yield, bronchus sign absent, No. (%)**	60/83 (72.3)	81/107 (78.5)	141/190 (74.2)	
**Sensitivity for malignancy (1 year), No. (%) [95% CI]**	99/121 (81.8) [73.9-87.7]	92/108 (85.2) [77.2-90.8]	191/229 (83.4) [77.9-88.0]	
**Sensitivity for malignancy (2 years), No. (%) [95% CI]**	99/127 (77.95) [69.9-84.3]	92/108 (85.2) [77.2-90.8]	191/235 (81.3) [75.7-86.1]	
**Sensitivity for malignancy (2 years) 1-2 cm, No. (%)**	56/74 (75.7)	63/76 (82.9)	19/150 (79.3)	.31
**Sensitivity for malignancy (2 years) >2-3 cm, No. (%)**	43/53 (81.1)	29/32 (90.6)	72/85 (84.7)	
**Sensitivity for malignancy (2 years), bronchus sign present, No. (%)**	45/58 (77.6)	28/32 (87.5)	73/90 (81.1)	.98
**Sensitivity for malignancy (2 years), bronchus sign absent, No. (%)**	53/68 (77.9)	64/76 (84.2)	117/144 (81.3)	
**Prevalence of malignancy, No. (%)**	127/150 (84.7)	108/155 (69.7)	235/305 (77.05)	

Abbreviations: ATS/ACCP, American Thoracic Society/American College of Chest Physicians; CI, confidence interval.

Prevalence of cancer in this population was 235/305 (77.11%) and varied across centers (62%-88%; *P* = .02). Of the primary lung cancers diagnosed with ssRAB, approximately 68.2% were classified as stage IA using the American Joint Committee on Cancer (AJCC) 8th edition staging system. The probability of malignancy in patients with a nondiagnostic result was 43/74 (59.5% [95% CI, 47.4%-70.7%]).

Diagnostic yield using the intermediate study definition was 77.5% (95% CI, 72.4%-81.8%) and varied from 62% to 88% across centers (*P* = .89). Diagnostic yield according to the ATS/ACCP recommendation was 74.1% (95% CI, 68.8%-78.9%), ranging from 60% to 88% (*P* = .58, Table 3). One subject with necrotizing granulomas considered diagnostic per the ATS/ACCP guidelines was diagnosed with a malignant condition in long-term follow-up.

A post hoc analysis was performed to assess the diagnostic accuracy using the methodology from the aforementioned ATS/ACCP recommendation. Diagnostic accuracy, calculated as TP malignant plus TN specific benign divided by all cases from ATS/ACCP-defined specific benign cases relative to the sum of all cases performed, was 225/305 (73.8% [95% CI, 68.45%-78.6%]). Details are provided in the online supplement. Multilevel analysis (Table 4) identified the following variables as being associated with higher sensitivity for malignancy: male sex (OR, 2.58; *P* = .023), nodule diameter (OR, 1.07 per mm increase; *P* = .04), and each subsequent case performed by the proceduralist (OR, 1.04; *P* = .01). Conversely, lower lobe location (OR, 0.38; *P* = .02), semi-solid nodules (OR, 0.36; *P* = .04), and higher body mass index (BMI) (OR, 0.95 per unit increase [kg/m^2^]; *P* = .031) were associated with lower sensitivity for malignancy. In post hoc analysis, malignant nodules in the lowest quartile of size (≤4 mm) had a lower sensitivity than nodules in the top 3 quartiles (sensitivity of 71.8% vs 84.5% vs 87.0% vs 85%, respectively, *P* = .046; see online ­supplement). Center when treated as a random effect was not a significant factor (*P* = .75).

**Table 4:** Multilevel models.

**Diagnostic yield (ATS/ACCP) with site and physician as nested random effect** ^a^ ^,^ ^b^				
		Multivariate		
Variables selected for model	Reference	OR	(95% CI)	*P* value
**BMI**	By 1-unit increase	0.94	(0.90-0.98)	.002
**Distance from pleural wall**	By 1-mm increase	0.98	(0.96-1.0)	.10
**Lung lesion location, lower lobe**	Upper lobe	0.52	(0.3-0.89)	.02
**Semisolid nodule**	Solid	0.39	(0.19-0.78)	.01
**Largest nodule diameter**	By 1-mm increase	1.05	(1.0-1.11)	.06
**Number of cases performed 60 days prior to present case**	By 1-unit increase	1.03	(0.97-1.1)	.09

**2-year sensitivity for malignancy with site as random effect** ^c^ ^,^ ^d^				
		Multivariate		
**Variables selected for model** ^d^ ^,^ ^e^	Reference	OR	(95% CI)	*P* value
**Male**	Female	2.58	(1.17-6.09)	.023
**Lower lobe nodule location**	Upper lobe	0.38	(0.16-0.87)	.021
**Mixed lobe nodule location**	Upper lobe	0.50	(0.18-1.54)	.21
**Mixed nodule density**	Solid	1.68	(0.21-35.85)	.665
**Semisolid nodule density**	Solid	0.36	(0.14-0.96)	.04
**BMI**	By 1-unit increase	0.95	(0.9-1.0)	.03
**Largest nodule diameter (mm)**	By 1-mm increase	1.07	(1.0-1.16)	.045
**Investigator case number**	By 1-case increase	1.04	(1.01-1.08)	.0105

Abbreviations: ATS/ACCP, American Thoracic Society/American College of Chest Physicians; BMI, body mass index; CI, confidence interval; OR, odds ratio.

a Likelihood ratio test (LRT) on site and physician random effects: LRT = 0.8509 (degrees of freedom [df] = 1); *P* = .356.

b Variables were selected using stepwise logistic regression based on Akaike information criterion (AIC). Site and physician were excluded because they were forced into the model as random effects. Univariate results for all variables tested are provided in the online supplement for reference.

c LRT on random effects: LRT = 0.1033 (df = 1); *P* = .7479.

d Variables were selected using stepwise logistic regression based on AIC.

e Due to the small number of physicians in each site, site was modeled as a random effect in the multilevel model.

Multilevel analysis identified the following variables as being associated with higher diagnostic yield as defined by the ATS/ACCP criteria: nodule diameter (OR, 1.05 per mm increase; *P* = .06) and each additional case (1-unit increase) in the 60 days prior to the case performed (OR, 1.03; *P* = .09). Conversely, higher BMI (OR, 0.94 per unit increase [kg/m^2^]; *P* = .002), increased distance from pleura (OR, 0.98 per mm increase; *P* = .10), semi-solid nodules (OR, 0.39; *P* = .01), and lower lobe location (OR, 0.52; *P* = .02) were associated with lower diagnostic yield (Table 4). Center as a random effect failed to reach statistical significance for diagnostic yield (*P* = .36).

In a few cases multiple nodules were biopsied during the study procedures using ssRAB. The diagnostic yields above are reported at the patient level and include all nodules biopsied during the procedure. A post hoc analysis of sensitivity and diagnostic yield (both ATS/ACCP and intermediate criteria) was performed specific to the first nodule biopsied only and results were similar between both groups (see online supplement).

## Discussion

In this first multicenter, prospective, observational study of ssRAB, we report a sensitivity for malignancy of 81.3% in pulmonary nodules with a median size of 17 mm. The procedure was safe, with pneumothorax requiring intervention and airway bleeding occurring in 1.6% and 1.0% of cases, respectively. These results reflect the initial experience of 11 robotic-naive users with the first-generation ssRAB system.

Sensitivity for malignancy is a key metric for evaluating diagnostic tests for lung nodules.^15^^,^^17^ The ATS and ACCP recommend at least 12 months of follow-up to assess sensitivity, though supporting data are limited. Prior robotic bronchoscopy studies typically reported 1-year follow-up results. This study, the first to evaluate ssRAB with 2-year follow-up, observed a 2.1% decrease in sensitivity from year 1 (83.4%) to year 2 (81.3%) due to additional cancers detected in the second year. This finding suggests that the ATS/ACCP 12-month follow-up is reasonable but may slightly overestimate sensitivity.^15^ While sensitivity is focused on malignancy, peripheral nodule biopsies are a multidisease test and some patients will have a benign reference state. Diagnostic accuracy is a complimentary metric to sensitivity as it includes patients with a benign condition. Diagnostic accuracy using the ATS/ACCP benign specific definition for TN, 73.8%, is negligibly different from the ATS/ACCP yield of 74.1%, suggesting that the ATS/ACCP diagnostic yield may be a relatively accurate estimate of diagnostic accuracy without needing long-term follow-up.

To contextualize these results, we compared ssRAB with other multicenter bronchoscopy studies. The NAVIGATE trial, using nonrobotic EMN bronchoscopy, reported a 2-year sensitivity of 62.6% for nodules with a median size of 20 mm.^18^ The TARGET trial, using an EMN robotic-assisted-bronchoscopy (EMN-RAB) system, reported a 1-year sensitivity of 78.8% for nodules with a median size of 21 mm.^19^ The ssRAB sensitivities at 1 and 2 years (83.4% and 81.3%) in our study (PRECIsE) are comparable to those in TARGET. Prevalence of malignancy varied between NAVIGATE, TARGET, and PRECIsE (68% vs 64.1% vs 77%).

The high malignancy prevalence in PRECIsE influenced the posterior probability of cancer (pCA) for nondiagnostic biopsies. Previous RAB studies rarely reported pCA or provide contingency tables.^17^ Traditionally, pCA for nondiagnostic tests was estimated using the false omission rate (1 minus negative predictive value), which assumes a binary test. However, bronchoscopy is a multidisease diagnostic tool, rendering this approach flawed. Using false omission rate underestimates the true probability of malignancy in patients with nondiagnostic results.^20^ In PRECIsE, the pCA for nondiagnostic bronchoscopies was 58.1%, substantially higher than the false omission rate (39%). Thus, despite improvements in RAB sensitivity, aggressive follow-up of nondiagnostic robotic biopsies is warranted, particularly when pretest probability is high. Previous decision analyses suggest that aggressive follow-up is likely to be cost-effective as well.^8^

Diagnostic yield, unlike sensitivity, is less reliable for cross-study comparisons due to its dependence on malignancy prevalence and the distribution of other diseases.^17^^,^^20^ In the NAVIGATE trial, diagnostic yield per strict ATS/ACCP guidelines was 48.7% with a malignancy prevalence of 68%.^18^^,^^21^ In the TARGET trial, diagnostic yield was 61.6%, and in PRECIsE, it was 74.1%.^22^ Although ssRAB appears to be superior to EMN-RAB in TARGET based on diagnostic yield, this comparison is misleading. Sensitivity is a more robust metric for evaluating similarly sized and located nodules. The ssRAB sensitivity of 81.3% in PRECIsE is comparable to the 78.8% sensitivity of EMN-RAB in TARGET. Nonrobotic EMN sensitivity in NAVIGATE was 62.6%. These findings highlight the superiority of sensitivity over diagnostic yield for comparing bronchoscopy technologies across studies.

Center-level effects, critical for assessing the reproducibility of new technologies, were also evaluated. Multilevel analysis showed that sensitivity for malignancy was positively associated with larger nodule diameter, lower BMI, solid nodules, upper lobe location, and greater ssRAB experience. No significant center-level effects were detected for sensitivity, though the study was underpowered, making these findings hypothesis-generating. Center-level effects on diagnostic yield failed to reach statistical significance (*P* = .36), even though there were significant variations in malignancy prevalence, disease distributions, rapid onsite evaluation, cytologist/pathologist expertise, and workflows.

The safety profile of ssRAB aligns with prior bronchoscopy studies. PRECIsE reported pneumothorax and bleeding rates of 3.8% and 0.8%, respectively, across 365 subjects. NAVIGATE reported 4.7% pneumothorax and 2.7% bleeding; AQUIRE reported 1.7% pneumothorax and 0.17% bleeding; and TARGET reported 2.8% pneumothorax and 1% bleeding.^18^^,^^19^^,^^23^

Limitations include prolonged enrollment due to the COVID-19 pandemic, which delayed study completion. As an observational study, PRECIsE specified the patient population and tool sequence but did not control other procedural parameters to ensure pragmatic outcomes. Technological advancements, such as CBCT integration, and training programs implemented poststudy may enhance performance. The study was conducted in high-volume academic and community centers by bronchoscopists experienced in navigational bronchoscopy, potentially limiting generalizability. The use of sensitivity as the primary outcome, while providing benefits over diagnostic yield, is also challenging since 6 patients (2%) did not have complete follow-up. This limits the accuracy of sensitivity measurements. Our sensitivity analysis suggests that in a worst-case scenario, sensitivity could be as low as 79.3%, which is only 2% lower than the 81.3% 2-year sensitivity, which was our primary outcome.

In conclusion, this first prospective, multicenter study of ssRAB demonstrates high diagnostic sensitivity and safety for small pulmonary nodules. Despite improved sensitivity, nondiagnostic biopsies require vigilant follow-up due to a high posterior probability of malignancy. The study validates ATS/ACCP 12-month follow-up recommendations while quantifying the associated sensitivity overestimation that results. Multilevel modeling identified lower lobe location, semisolid density, smaller nodule size, higher BMI, and less operator experience as factors associated with reduced sensitivity. These findings highlight the value of multicenter studies in evaluating emerging bronchoscopy technologies.

## Supplementary Material

aaoag049_Supplementary_Data
