# Supplementary material for: The PRECIsE study: a prospective, multicenter study of shape-sensing robotic-assisted bronchoscopy with 2 years of follow-up
Source: Ann Am Thorac Soc. 2026 Mar 3;23(9):1360–8. doi: 10.1093/annalsats/aaoag049 (PMC13521439; doi:10.1093/annalsats/aaoag049)
Supplement: Supplementary file 1 [file aaoag049_supplementary_data.zip › Binder1.pdf]

ICMJE DISCLOSURE FORM

Date:_22-Dec-2025
Your Name: Roberto Casal
Manuscript Title: The PRECIsE Study: a prospective, multicenter study of shape-sensing robotic-assisted bronchoscopy with two years of follow-up
Manuscript number (if known): White-202506-660OC.R2

In the interest of transparency, we ask you to disclose all relationships/activities/interests listed below that are related to the content of your manuscript. "Related" means any relation with for-profit or not-for-profit third parties whose interests may be affected by the content of the manuscript. Disclosure represents a commitment to transparency and does not necessarily indicate a bias. If you are in doubt about whether to list a relationship/activity/interest, it is preferable that you do so.

The following questions apply to the author's relationships/activities/interests as they relate to the current manuscript only.

The author's relationships/activities/interests should be defined broadly. For example, if your manuscript pertains to the epidemiology of hypertension, you should declare all relationships with manufacturers of antihypertensive medication, even if that medication is not mentioned in the manuscript.

In item #1 below, report all support for the work reported in this manuscript without time limit. For all other items, the time frame for disclosure is the past 36 months.

		**Name all entities with whom you have this relationship or indicate none (add rows as needed)**	**Specifications/Comments (e.g., if payments were made to you or to your institution)**
**Time frame: Since the initial planning of the work**			
1	All support for the present manuscript (e.g., funding, provision of study materials, medical writing, article processing charges, etc.) **No time limit for this item.**	Intuitive Surgical	Clinical trial grant to institution
**Time frame: past 36 months**			
2	Grants or contracts from any entity (if not indicated in item #1 above).	Siemens	Research grant.
		Johnson and Johnson	Research grant.
3	Royalties or licenses	____None	
4	Consulting fees	Intuitive<br>Canon	

		Olympus	
		Johnson and Johnson	
5	Payment or honoraria for lectures, presentations, speakers bureaus, manuscript writing or educational events	____None	
6	Payment for expert testimony	____None	
7	Support for attending meetings and/or travel	____None	
8	Patents planned, issued or pending	____None	
9	Participation on a Data Safety Monitoring Board or Advisory Board	____None	
10	Leadership or fiduciary role in other board, society, committee or advocacy group, paid or unpaid	____None	
11	Stock or stock options	____None	
12	Receipt of equipment, materials, drugs, medical writing, gifts or other services	____None	
13	Other financial or non-financial interests	____None	

Please place an "X" next to the following statement to indicate your agreement:

X I certify that I have answered every question and have not altered the wording of any of the questions on this form.

ICMJE DISCLOSURE FORM

Date:_22-Dec-2025
Your Name: Javier Diaz-Mendoza
Manuscript Title: The PRECIsE Study: a prospective, multicenter study of shape-sensing robotic-assisted bronchoscopy with two years of follow-up
Manuscript number (if known): White-202506-660OC.R2

In the interest of transparency, we ask you to disclose all relationships/activities/interests listed below that are related to the content of your manuscript. "Related" means any relation with for-profit or not-for-profit third parties whose interests may be affected by the content of the manuscript. Disclosure represents a commitment to transparency and does not necessarily indicate a bias. If you are in doubt about whether to list a relationship/activity/interest, it is preferable that you do so.

The following questions apply to the author's relationships/activities/interests as they relate to the current manuscript only.

The author's relationships/activities/interests should be defined broadly. For example, if your manuscript pertains to the epidemiology of hypertension, you should declare all relationships with manufacturers of antihypertensive medication, even if that medication is not mentioned in the manuscript.

In item #1 below, report all support for the work reported in this manuscript without time limit. For all other items, the time frame for disclosure is the past 36 months.

		**Name all entities with whom you have this relationship or indicate none (add rows as needed)**	**Specifications/Comments (e.g., if payments were made to you or to your institution)**
		**Time frame: Since the initial planning of the work**	
1	All support for the present manuscript (e.g., funding, provision of study materials, medical writing, article processing charges, etc.) **No time limit for this item.**	Intuitive Surgical	Clinical trial grant to institution
		**Time frame: past 36 months**	
2	Grants or contracts from any entity (if not indicated in item #1 above).	____None	
3	Royalties or licenses	____None	
4	Consulting fees	____None	

5	Payment or honoraria for lectures, presentations, speakers bureaus, manuscript writing or educational events	____None	
6	Payment for expert testimony	____None	
7	Support for attending meetings and/or travel	____None	
8	Patents planned, issued or pending	____None	
9	Participation on a Data Safety Monitoring Board or Advisory Board	____None	
10	Leadership or fiduciary role in other board, society, committee or advocacy group, paid or unpaid	____None	
11	Stock or stock options	____None	
12	Receipt of equipment, materials, drugs, medical writing, gifts or other services	____None	
13	Other financial or non-financial interests	____None	

Please place an "X" next to the following statement to indicate your agreement:

__X_ I certify that I have answered every question and have not altered the wording of any of the questions on this form.

ICMJE DISCLOSURE FORM

Date:_22-Dec-2025
Your Name: Sebastian Fernandez-Bussy
Manuscript Title: The PRECIsE Study: a prospective, multicenter study of shape-sensing robotic-assisted bronchoscopy with two years of follow-up
Manuscript number (if known): White-202506-660OC.R2

In the interest of transparency, we ask you to disclose all relationships/activities/interests listed below that are related to the content of your manuscript. "Related" means any relation with for-profit or not-for-profit third parties whose interests may be affected by the content of the manuscript. Disclosure represents a commitment to transparency and does not necessarily indicate a bias. If you are in doubt about whether to list a relationship/activity/interest, it is preferable that you do so.

The following questions apply to the author's relationships/activities/interests as they relate to the current manuscript only.

The author's relationships/activities/interests should be defined broadly. For example, if your manuscript pertains to the epidemiology of hypertension, you should declare all relationships with manufacturers of antihypertensive medication, even if that medication is not mentioned in the manuscript.

In item #1 below, report all support for the work reported in this manuscript without time limit. For all other items, the time frame for disclosure is the past 36 months.

		**Name all entities with whom you have this relationship or indicate none (add rows as needed)**	**Specifications/Comments (e.g., if payments were made to you or to your institution)**
		**Time frame: Since the initial planning of the work**	
1	All support for the present manuscript (e.g., funding, provision of study materials, medical writing, article processing charges, etc.) **No time limit for this item.**	Intuitive Surgical	Clinical trial grant to institution
		**Time frame: past 36 months**	
2	Grants or contracts from any entity (if not indicated in item #1 above).	_X___None	
3	Royalties or licenses	__X__None	
4	Consulting fees	_____None	Consulting fees paid by Intuitive Surgical

5	Payment or honoraria for lectures, presentations, speakers bureaus, manuscript writing or educational events	____None	Honorarium payment for presentations paid by Intuitive Surgical
6	Payment for expert testimony	__X__None	
7	Support for attending meetings and/or travel	____None	Travel costs paid by Intuitive Surgical
8	Patents planned, issued or pending	__X__None	
9	Participation on a Data Safety Monitoring Board or Advisory Board	_X___None	
10	Leadership or fiduciary role in other board, society, committee or advocacy group, paid or unpaid	__X__None	
11	Stock or stock options	_X___None	
12	Receipt of equipment, materials, drugs, medical writing, gifts or other services	X____None	
13	Other financial or non-financial interests	__X__None	

Please place an "X" next to the following statement to indicate your agreement:

_X__ I certify that I have answered every question and have not altered the wording of any of the questions on this form.

ICMJE DISCLOSURE FORM

**Date:**	2/19/2026
**Your Name:**	Erik E. Folch, M.D.
**Manuscript Title:**	The PRECIsE Study: a prospective, multicenter study of shape-sensing robotic-assisted bronchoscopy with two years of follow-up
**Manuscript Number (if known):**	White-202506-660OC.R2

In the interest of transparency, we ask you to disclose all relationships/activities/interests listed below that are related to the content of your manuscript. "Related" means any relation with for-profit or not-for-profit third parties whose interests may be affected by the content of the manuscript. Disclosure represents a commitment to transparency and does not necessarily indicate a bias. If you are in doubt about whether to list a relationship/activity/interest, it is preferable that you do so.

The author's relationships/activities/interests should be defined broadly. For example, if your manuscript pertains to the epidemiology of hypertension, you should declare all relationships with manufacturers of antihypertensive medication, even if that medication is not mentioned in the manuscript.

In item #1 below, report all support for the work reported in this manuscript without time limit. For all other items, the time frame for disclosure is the past 36 months.

		**Name all entities with whom you have this relationship or indicate none (add rows as needed)**	**Specifications/Comments (e.g., if payments were made to you or to your institution)**
**Time frame: Since the initial planning of the work**			
1	All support for the present manuscript (e.g., funding, provision of study materials, medical writing, article processing charges, etc.) **No time limit for this item.**	☐ None	
		Intuitive Surgical, Inc.	Research funding to institution in support of this study. No direct payments were made for this present manuscript
			Click the tab key to add additional rows.
**Time frame: past 36 months**			
**2**	Grants or contracts from any entity (if not indicated in item #1 above).	☒ **None**	
**3**	Royalties or licenses	☒ **None**	

		Name all entities with whom you have this relationship or indicate none (add rows as needed)	Specifications/Comments (e.g., if payments were made to you or to your institution)
**4**	Consulting fees	☐ **None**	
		Intuitive Surgical, Inc.	No direct fees were received during the conduct of this study
		Boston Scientific	Consulting fees
		Medtronic	Consulting fees
		Cook	Consulting fees
		Siemens	Consulting fees
		Pinnacle Biologics	Consulting fees
		Zidan	Consulting Fees
**5**	Payment or honoraria for lectures, presentations, speakers bureaus, manuscript writing or educational events	☒ **None**	
**6**	Payment for expert testimony	☒ **None**	
**7**	Support for attending meetings and/or travel	☐ **None**	
**8**	Patents planned, issued or pending	☒ **None**	
**9**	Participation on a Data Safety Monitoring Board or Advisory Board	☐ **None**	
		Nanotherm	Scientific Advisor
**10**	Leadership or fiduciary role in other board, society, committee or advocacy group, paid or unpaid	☒ **None**	

		Name all entities with whom you have this relationship or indicate none (add rows as needed)	Specifications/Comments (e.g., if payments were made to you or to your institution)
**11**	Stock or stock options	☒ **None**	
**12**	Receipt of equipment, materials, drugs, medical writing, gifts or other services	☒ **None**	
**13**	Other financial or non-financial interests	☒ **None**	

Please place an "X" next to the following statement to indicate your agreement:

☒ I certify that I have answered every question and have not altered the wording of any of the questions on this form.

ICMJE DISCLOSURE FORM

Date:_22-Dec-2025
Your Name: Colleen Keyes
Manuscript Title: The PRECIsE Study: a prospective, multicenter study of shape-sensing robotic-assisted bronchoscopy with two years of follow-up
Manuscript number (if known): White-202506-660OC.R2

In the interest of transparency, we ask you to disclose all relationships/activities/interests listed below that are related to the content of your manuscript. "Related" means any relation with for-profit or not-for-profit third parties whose interests may be affected by the content of the manuscript. Disclosure represents a commitment to transparency and does not necessarily indicate a bias. If you are in doubt about whether to list a relationship/activity/interest, it is preferable that you do so.

The following questions apply to the author's relationships/activities/interests as they relate to the current manuscript only.

The author's relationships/activities/interests should be defined broadly. For example, if your manuscript pertains to the epidemiology of hypertension, you should declare all relationships with manufacturers of antihypertensive medication, even if that medication is not mentioned in the manuscript.

In item #1 below, report all support for the work reported in this manuscript without time limit. For all other items, the time frame for disclosure is the past 36 months.

		**Name all entities with whom you have this relationship or indicate none (add rows as needed)**	**Specifications/Comments (e.g., if payments were made to you or to your institution)**
**Time frame: Since the initial planning of the work**			
1	All support for the present manuscript (e.g., funding, provision of study materials, medical writing, article processing charges, etc.) **No time limit for this item.**	Intuitive Surgical	Clinical trial grant to institution
**Time frame: past 36 months**			
2	Grants or contracts from any entity (if not indicated in item #1 above).	National Institutes of Health	Co-investigator on grants to institution, research using optical coherence tomography in ILD patients
		Department of Defense	Co-investigator on grant to institution, research on optical coherence tomography in post-COVID patients
		Boehringer Ingelheim Pharmaceuticals, Inc.	Co-investigator on grant to institution, research on optical coherence tomography in post-COVID patients

3	Royalties or licenses	__X__None	
4	Consulting fees	__X__None	
5	Payment or honoraria for lectures, presentations, speakers bureaus, manuscript writing or educational events	Intuitive Surgical	Educational event on robotic bronchoscopy case review on patients who were not study subjects
		Cook Medical, Inc	Educational event on percutaneous tracheostomy
6	Payment for expert testimony	__X__None	
7	Support for attending meetings and/or travel	Intuitive Surgical	Women in da Vinci Surgery 2025, WiIP mentors summit
8	Patents planned, issued or pending	__X__None	
9	Participation on a Data Safety Monitoring Board or Advisory Board	__X__None	
10	Leadership or fiduciary role in other board, society, committee or advocacy group, paid or unpaid	AABIP	Unpaid position on board of advisors
		Women in IP	Unpaid position on executive board
11	Stock or stock options	__X__None	
12	Receipt of equipment, materials, drugs, medical writing, gifts or other services	__X__None	
13	Other financial or non-financial interests	__X__None	

Please place an "X" next to the following statement to indicate your agreement:

_X__ I certify that I have answered every question and have not altered the wording of any of the questions on this form.

ICMJE DISCLOSURE FORM

Date:_22-Dec-2025
Your Name: Adnan Majid
Manuscript Title: The PRECIsE Study: a prospective, multicenter study of shape-sensing robotic-assisted bronchoscopy with two years of follow-up
Manuscript number (if known): White-202506-660OC.R2

In the interest of transparency, we ask you to disclose all relationships/activities/interests listed below that are related to the content of your manuscript. "Related" means any relation with for-profit or not-for-profit third parties whose interests may be affected by the content of the manuscript. Disclosure represents a commitment to transparency and does not necessarily indicate a bias. If you are in doubt about whether to list a relationship/activity/interest, it is preferable that you do so.

The following questions apply to the author's relationships/activities/interests as they relate to the current manuscript only.

The author's relationships/activities/interests should be defined broadly. For example, if your manuscript pertains to the epidemiology of hypertension, you should declare all relationships with manufacturers of antihypertensive medication, even if that medication is not mentioned in the manuscript.

In item #1 below, report all support for the work reported in this manuscript without time limit. For all other items, the time frame for disclosure is the past 36 months.

		**Name all entities with whom you have this relationship or indicate none (add rows as needed)**	**Specifications/Comments (e.g., if payments were made to you or to your institution)**
		**Time frame: Since the initial planning of the work**	
1	All support for the present manuscript (e.g., funding, provision of study materials, medical writing, article processing charges, etc.) **No time limit for this item.**	Intuitive Surgical	Clinical trial grant to institution
		**Time frame: past 36 months**	
2	Grants or contracts from any entity (if not indicated in item #1 above).	____None	
		Olympus	Clinical trial grant to institution
		CSA Medical	Clinical trial grant to institution
		Galvanize	Clinical trial grant to institution
		Apreo Health	Clinical trial grant to institution
		PulmAir	Clinical trial grant to institution
		PulmonX	Clinical trial grant to institution
		NIH	Clinical trial grant to institution

3	Royalties or licenses	___X_None	
4	Consulting fees	____None	
		J&J	Consultant
5	Payment or honoraria for lectures, presentations, speakers bureaus, manuscript writing or educational events	____None	
		Olympus	Educational Consultant
		PulmonX<br>Steris<br>Intuitive Surgical<br>Pinnacle Biologics	
6	Payment for expert testimony	__x__None	
7	Support for attending meetings and/or travel	__x__None	
8	Patents planned, issued or pending	__x__None	
9	Participation on a Data Safety Monitoring Board or Advisory Board	_x___None	
10	Leadership or fiduciary role in other board, society, committee or advocacy group, paid or unpaid	____None	
		AABIP	Board of Directors
		JOBIP	Editorial Board
11	Stock or stock options	__X__None	
12	Receipt of equipment, materials, drugs, medical writing, gifts or other services	__X__None	
13	Other financial or non-financial interests	__x__None	

Please place an "X" next to the following statement to indicate your agreement:

_X__ I certify that I have answered every question and have not altered the wording of any of the questions on this form.

ICMJE DISCLOSURE FORM

Date: 12/19/2025
Your Name: David Ost, MD
Manuscript Title: The PRECIsE Study: a prospective, multicenter study of shape-sensing robotic-assisted bronchoscopy with two years of follow-up
Manuscript number (if known): White-202506-660OC.R2

In the interest of transparency, we ask you to disclose all relationships/activities/interests listed below that are related to the content of your manuscript. "Related" means any relation with for-profit or not-for-profit third parties whose interests may be affected by the content of the manuscript. Disclosure represents a commitment to transparency and does not necessarily indicate a bias. If you are in doubt about whether to list a relationship/activity/interest, it is preferable that you do so.

The following questions apply to the author's relationships/activities/interests as they relate to the current manuscript only.

The author's relationships/activities/interests should be defined broadly. For example, if your manuscript pertains to the epidemiology of hypertension, you should declare all relationships with manufacturers of antihypertensive medication, even if that medication is not mentioned in the manuscript.

In item #1 below, report all support for the work reported in this manuscript without time limit. For all other items, the time frame for disclosure is the past 36 months.

		**Name all entities with whom you have this relationship or indicate none (add rows as needed)**	**Specifications/Comments (e.g., if payments were made to you or to your institution)**
		**Time frame: Since the initial planning of the work**	
1	All support for the present manuscript (e.g., funding, provision of study materials, medical writing, article processing charges, etc.) **No time limit for this item.**	Intuitive Surgical	Clinical trial grant to institution (MDACC)
		**Time frame: past 36 months**	
2	Grants or contracts from any entity (if not indicated in item #1 above).	Becton Dickensen	Consultant
		Johnson and Johnson	Consultant
3	Royalties or licenses	Elsevier	Royalties on textbook, "Practical Guide to Interventional Pulmonology
4	Consulting fees	Intuitive Surgical	consulting

5	Payment or honoraria for lectures, presentations, speakers bureaus, manuscript writing or educational events	____None	
6	Payment for expert testimony	____None	
7	Support for attending meetings and/or travel	____None	
8	Patents planned, issued or pending	____None	
9	Participation on a Data Safety Monitoring Board or Advisory Board	____None	
10	Leadership or fiduciary role in other board, society, committee or advocacy group, paid or unpaid	____None	
11	Stock or stock options	____None	
12	Receipt of equipment, materials, drugs, medical writing, gifts or other services	____None	
13	Other financial or non-financial interests	____None	

Please place an "X" next to the following statement to indicate your agreement:

_X__ I certify that I have answered every question and have not altered the wording of any of the questions on this form.

ICMJE DISCLOSURE FORM

Date:_22-Dec-2025
Your Name: Mihir Parikh
Manuscript Title: The PRECIsE Study: a prospective, multicenter study of shape-sensing robotic-assisted bronchoscopy with two years of follow-up
Manuscript number (if known): White-202506-660OC.R2

In the interest of transparency, we ask you to disclose all relationships/activities/interests listed below that are related to the content of your manuscript. "Related" means any relation with for-profit or not-for-profit third parties whose interests may be affected by the content of the manuscript. Disclosure represents a commitment to transparency and does not necessarily indicate a bias. If you are in doubt about whether to list a relationship/activity/interest, it is preferable that you do so.

The following questions apply to the author's relationships/activities/interests as they relate to the current manuscript only.

The author's relationships/activities/interests should be defined broadly. For example, if your manuscript pertains to the epidemiology of hypertension, you should declare all relationships with manufacturers of antihypertensive medication, even if that medication is not mentioned in the manuscript.

In item #1 below, report all support for the work reported in this manuscript without time limit. For all other items, the time frame for disclosure is the past 36 months.

		Name all entities with whom you have this relationship or indicate none (add rows as needed)	Specifications/Comments (e.g., if payments were made to you or to your institution)
		**Time frame: Since the initial planning of the work**	
1	All support for the present manuscript (e.g., funding, provision of study materials, medical writing, article processing charges, etc.) **No time limit for this item.**	Intuitive Surgical	Clinical trial grant to institution
		**Time frame: past 36 months**	
2	Grants or contracts from any entity (if not indicated in item #1 above).	_X_None	
3	Royalties or licenses	_X_None	
4	Consulting fees	____None	
		Intuitive Surgical	Educational Consulting

5	Payment or honoraria for lectures, presentations, speakers bureaus, manuscript writing or educational events	X None	
6	Payment for expert testimony	X None	
7	Support for attending meetings and/or travel	X None	
8	Patents planned, issued or pending	X None	
9	Participation on a Data Safety Monitoring Board or Advisory Board	X None	
10	Leadership or fiduciary role in other board, society, committee or advocacy group, paid or unpaid	X None	
11	Stock or stock options	X None	
12	Receipt of equipment, materials, drugs, medical writing, gifts or other services	X None	
13	Other financial or non-financial interests	X None	

Please place an "X" next to the following statement to indicate your agreement:

X **I certify that I have answered every question and have not altered the wording of any of the questions on this form.**

ICMJE DISCLOSURE FORM

Date:_22-Dec-2025
Your Name: Janani Reisenauer
Manuscript Title: The PRECIsE Study: a prospective, multicenter study of shape-sensing robotic-assisted bronchoscopy with two years of follow-up
Manuscript number (if known): White-202506-660OC.R2

In the interest of transparency, we ask you to disclose all relationships/activities/interests listed below that are related to the content of your manuscript. "Related" means any relation with for-profit or not-for-profit third parties whose interests may be affected by the content of the manuscript. Disclosure represents a commitment to transparency and does not necessarily indicate a bias. If you are in doubt about whether to list a relationship/activity/interest, it is preferable that you do so.

The following questions apply to the author's relationships/activities/interests as they relate to the <u>current manuscript only</u>.

The author's relationships/activities/interests should be <u>defined broadly</u>. For example, if your manuscript pertains to the epidemiology of hypertension, you should declare all relationships with manufacturers of antihypertensive medication, even if that medication is not mentioned in the manuscript.

In item #1 below, report all support for the work reported in this manuscript without time limit. For all other items, the time frame for disclosure is the past 36 months.

		**Name all entities with whom you have this relationship or indicate none (add rows as needed)**	**Specifications/Comments (e.g., if payments were made to you or to your institution)**
**Time frame: Since the initial planning of the work**			
1	All support for the present manuscript (e.g., funding, provision of study materials, medical writing, article processing charges, etc.) **No time limit for this item.**	Intuitive Surgical	Clinical trial grant to institution
**Time frame: past 36 months**			
2	Grants or contracts from any entity (if not indicated in item #1 above).	____None	
3	Royalties or licenses	____None	
4	Consulting fees	____None	

5	Payment or honoraria for lectures, presentations, speakers bureaus, manuscript writing or educational events	____None	
6	Payment for expert testimony	____None	
7	Support for attending meetings and/or travel	____None	
8	Patents planned, issued or pending	____None	
9	Participation on a Data Safety Monitoring Board or Advisory Board	____None	
10	Leadership or fiduciary role in other board, society, committee or advocacy group, paid or unpaid	____None	
11	Stock or stock options	____None	
12	Receipt of equipment, materials, drugs, medical writing, gifts or other services	____None	
13	Other financial or non-financial interests	____None	

Please place an "X" next to the following statement to indicate your agreement:

_x__ I certify that I have answered every question and have not altered the wording of any of the questions on this form.

ICMJE DISCLOSURE FORM

Date: 12/22/2025
Your Name: Michael Simoff, MD
Manuscript Title: The PRECIsE Study: a prospective, multicenter study of shape-sensing robotic-assisted bronchoscopy with two years of follow-up
Manuscript number (if known): White-202506-660OC.R2

In the interest of transparency, we ask you to disclose all relationships/activities/interests listed below that are related to the content of your manuscript. "Related" means any relation with for-profit or not-for-profit third parties whose interests may be affected by the content of the manuscript. Disclosure represents a commitment to transparency and does not necessarily indicate a bias. If you are in doubt about whether to list a relationship/activity/interest, it is preferable that you do so.

The following questions apply to the author's relationships/activities/interests as they relate to the <u>current manuscript only</u>.

The author's relationships/activities/interests should be <u>defined broadly</u>. For example, if your manuscript pertains to the epidemiology of hypertension, you should declare all relationships with manufacturers of antihypertensive medication, even if that medication is not mentioned in the manuscript.

In item #1 below, report all support for the work reported in this manuscript without time limit. For all other items, the time frame for disclosure is the past 36 months.

		**Name all entities with whom you have this relationship or indicate none (add rows as needed)**	**Specifications/Comments (e.g., if payments were made to you or to your institution)**
		**Time frame: Since the initial planning of the work**	
1	All support for the present manuscript (e.g., funding, provision of study materials, medical writing, article processing charges, etc.) **No time limit for this item.**	Intuitive Surgical	Clinical trial grant to institution (HFH)
		**Time frame: past 36 months**	
2	Grants or contracts from any entity (if not indicated in item #1 above).	Intuitive Surgical	Last 36 months Associate Medical Officer
3	Royalties or licenses	____None	
4	Consulting fees	Gongwin Biopharm	Consulting

5	Payment or honoraria for lectures, presentations, speakers bureaus, manuscript writing or educational events	____None	
6	Payment for expert testimony	____None	
7	Support for attending meetings and/or travel	____None	
8	Patents planned, issued or pending	____None	
9	Participation on a Data Safety Monitoring Board or Advisory Board	____None	
10	Leadership or fiduciary role in other board, society, committee or advocacy group, paid or unpaid	____None	
11	Stock or stock options	Intuitive Surgical	I do have stock as a Medical Officer. But at the time of the study, I did not have an financial relations with Intuitive surgical except the study and occasional speaking.
12	Receipt of equipment, materials, drugs, medical writing, gifts or other services	____None	
13	Other financial or non-financial interests	____None	

Please place an "X" next to the following statement to indicate your agreement:

__X_ I certify that I have answered every question and have not altered the wording of any of the questions on this form.
