# Supplementary material for: The PRECIsE study: a prospective, multicenter study of shape-sensing robotic-assisted bronchoscopy with 2 years of follow-up
Source: Ann Am Thorac Soc. 2026 Mar 3;23(9):1360–8. doi: 10.1093/annalsats/aaoag049 (PMC13521439; doi:10.1093/annalsats/aaoag049)
Supplement: Supplementary file 1 [file aaoag049_supplementary_data.zip › White-202506-660OC-PRECISE_Supplementary Table 1.docx]

**Supplementary Table 1 – Diagnostic Contingency Table**

| **ssRAB Biopsy result ↓** | | **Malignant Reference State** | | | | | | | | **Benign Reference State** | | | | | | | | | | | | | | | | | |  |
| --- | --- | --- | --- | --- | --- | --- | --- | --- | --- | --- | --- | --- | --- | --- | --- | --- | --- | --- | --- | --- | --- | --- | --- | --- | --- | --- | --- | --- |
|  |  | NSCLC | SCLC | Carcinoid | Metastatic cancer to the lung | Lymphoma | Neuroendocrine carcinoma | MALT | Cancer - not specified or Other Primary Lung | Aspergillus/ Aspergillosis | Mycobacteria  Mycobacteria avium complex  Mycobacterium simiae | Histo-plasmosis | Organizing or Cryptogenic Organizing Pneumonia | Pulmonary Hamartoma | Amyloid | Pseudo-monas | Bacteria, viral, fungal or other infectious agent | Sarcoidosis | Extramedullary hematopoesis | Benign hyperplasia / lymphocytes | Fibro-elastosis | Benign metastasizing leiomyoma | Pulmonary fibrosis and emphysema | Follow-up Tissue biopsy confirmed no malignancy | Radiographic Stability | Disappeared / decreased during observation | Lost to Follow-up | Total |
| **Malignant** | Non-small cell lung cancer | 147 |  |  | 1 |  |  |  |  |  |  |  |  |  |  |  |  |  |  |  |  |  |  |  |  |  |  | 148 |
|  | Small cell lung cancer |  | 8 |  |  |  |  |  |  |  |  |  |  |  |  |  |  |  |  |  |  |  |  |  |  |  |  | 8 |
|  | Carcinoid |  |  | 8 |  |  |  |  |  |  |  |  |  |  |  |  |  |  |  |  |  |  |  |  |  |  |  | 8 |
|  | Lymphoma |  |  |  |  | 2 |  |  |  |  |  |  |  |  |  |  |  |  |  |  |  |  |  |  |  |  |  | 2 |
|  | Neuroendocrine carcinoma |  |  |  |  |  | 3 |  |  |  |  |  |  |  |  |  |  |  |  |  |  |  |  |  |  |  |  | 3 |
|  | Metastatic cancer to the lung |  |  |  | 22 |  |  |  |  |  |  |  |  |  |  |  |  |  |  |  |  |  |  |  |  |  |  | 22 |
| **ATS/ACCP Specific Benign** | Aspergillus^a^ |  |  |  |  |  |  |  |  | 7 |  |  |  |  |  |  |  |  |  |  |  |  |  |  |  |  |  | 7 |
|  | Pyrenophora species /Marielliottia dematoididea ^a^ |  |  |  |  |  |  |  |  |  |  |  |  |  |  |  |  |  |  |  |  |  |  |  |  |  | 1 | 1 |
|  | Mycobacteria intracellulare |  |  |  |  |  |  |  |  |  | 3^b^ |  |  |  |  |  |  |  |  |  |  |  |  |  |  |  |  | 3 |
|  | Granuloma with positive cultures |  |  |  |  |  |  |  |  |  | 1^b^ |  |  |  |  |  |  |  |  |  |  |  |  |  |  |  |  | 1 |
|  | Granuloma with acid fast bacilli |  |  |  |  |  |  |  |  |  | 1^c^ |  |  |  |  |  |  |  |  |  |  |  |  |  |  |  |  | 1 |
|  | Granulomas w/out growth of an organism |  |  |  | 1^k (1)^ |  |  |  |  |  |  |  |  |  |  |  |  | 1 |  |  |  |  |  |  | 3 | 1 |  | 6 |
|  | Organizing Pneumonia |  |  |  |  |  |  |  |  |  |  |  | 1 |  |  |  |  |  |  |  |  |  |  |  | 1 | 3 |  | 5 |
|  | Lymphoeosinophilic inflammation, organizing fibrinous exudate |  |  |  |  |  |  |  |  |  |  |  |  |  |  |  |  |  |  |  |  |  |  |  | 1 |  |  | 1 |
|  | Histoplasmosis |  |  |  |  |  |  |  |  |  |  | 2 |  |  |  |  |  |  |  |  |  |  |  |  |  |  |  | 2 |
|  | Hamartoma |  |  |  |  |  |  |  |  |  |  |  |  | 1 |  |  |  |  |  |  |  |  |  |  |  |  |  | 1 |
|  | Pseudomonas |  |  |  |  |  |  |  |  |  |  |  |  |  |  | 1 |  |  |  |  |  |  |  |  |  |  |  | 1 |
|  | Bacterial infection - *Streptococcus anginosus* |  |  |  |  |  |  |  |  |  |  |  |  |  |  |  | 1 |  |  |  |  |  |  |  |  |  |  | 1 |
|  | Fungal infection (type unknown) |  |  |  |  |  |  |  |  |  |  |  |  |  |  |  |  |  |  |  |  |  |  |  |  | 1 |  | 1 |
|  | Extramedullary hematopoesis |  |  |  |  |  |  |  |  |  |  |  |  |  |  |  |  |  | 1^j^ |  |  |  |  |  |  |  |  | 1 |
|  | Fibroelastic scar |  |  |  |  |  |  |  |  |  |  |  |  |  |  |  |  |  |  |  |  |  |  |  | 1^m^ |  |  | 1 |
|  | Fibroelastosis |  |  |  |  |  |  |  |  |  |  |  |  |  |  |  |  |  |  |  | 1 |  |  |  | 1 |  |  | 2 |
| **Non-diagnostic (ATS/ACCP)**  **Diagnostic – Benign (Intermediate)** | Fibrosis |  |  |  |  |  |  |  |  |  |  |  | 1 |  |  |  |  |  |  |  |  |  |  |  |  |  |  | 1 |
|  | Fibroadipose tissue (suspected hamartoma) ^a^ |  |  |  |  |  |  |  |  |  |  |  |  |  |  |  |  |  |  |  |  |  |  |  |  |  | 1 | 1 |
|  | Lymphoid Tissue w/ polymorphouse lymphocytes (lymph node) |  |  |  |  |  |  |  |  |  |  |  |  |  |  |  |  |  |  | 1^g^ |  |  |  |  |  |  |  | 1 |
|  | Mucocele |  |  |  |  |  |  |  |  |  |  |  |  |  |  |  |  |  |  |  |  |  |  |  |  | 1^i^ |  | 1 |
|  | Necroinflammatory tissue/debris^a^ |  |  |  |  |  |  |  |  |  |  |  |  |  |  |  | 1^e^ |  |  |  |  |  |  |  |  |  | 1 | 2 |
|  | Fungal infection - candida species |  |  |  |  |  |  |  |  |  |  |  |  |  |  |  | 1^f^ |  |  |  |  |  |  |  |  |  |  | 1 |
| **Non-Diagnostic** | Fibrosis & chronic inflammation |  |  |  |  |  |  |  | 1 ^k(1),l^ |  |  |  |  |  |  |  |  |  |  |  |  |  |  |  |  |  |  | 1 |
|  | Atypical mucinous epithelial cells - Suspicious for mucinous adenocarcinoma | 1 |  |  |  |  |  |  |  |  |  |  |  |  |  |  |  |  |  |  |  |  |  |  |  |  |  | 1 |
|  | Chronic Inflammation ^a^ |  |  |  |  |  |  |  |  |  |  |  |  |  |  |  |  |  |  |  |  |  |  |  |  |  | 1 | 1 |
|  | Presence of macrophages, leukocytes, parenchyma and/or epithelial cells only^a^ | 7 |  | 1 | 2 |  |  |  | 1^l^ |  | 1^d^ |  |  | 1 |  |  |  |  |  |  |  | 1^h^ | 1 ^h^ |  | 3 | 1 | 1 | 20 |
|  | Fibrinous material w/extensive mucin |  |  |  |  |  |  |  |  |  |  |  |  |  |  |  |  |  |  |  |  |  |  |  |  | 1 |  | 1 |
|  | Atypical cells | 1 |  |  | 1 |  |  |  |  |  |  |  |  |  |  |  |  |  |  |  |  |  |  |  |  |  |  | 2 |
|  | Reactive squamous metaplasia | 1 |  |  |  |  |  |  |  |  |  |  |  |  |  |  |  |  |  |  |  |  |  |  |  |  |  | 1 |
|  | Acute inflammation w/out granulomas, no infectious agent grown | 3 |  |  | 1 |  |  | 1 | 1^o^ |  |  |  |  |  |  |  |  |  |  |  |  |  |  |  | 2 | 3 |  | 11 |
|  | Other* | 1 ^k(1)^ |  |  | 1 ^k(1)^ |  |  |  |  |  |  |  |  |  |  |  |  |  |  |  |  |  |  |  |  |  |  | 2 |
|  | Non-specific normal lung tissue ^a^ | 8 ^k(1)^ |  | 1 | 2 ^k(1)^ |  | 1 |  | 1^o^ |  |  |  | 1 |  | 1 |  | 1 |  |  |  |  |  |  | 1 | 5 | 1 | 1 | 24 |
|  | Blood only | 1 |  |  |  |  | 1 |  | 1 |  |  |  |  |  |  |  |  |  |  |  |  |  |  |  | 1 | 1 |  | 5 |
|  | Biopsy Not Completed | 2 |  |  |  |  |  |  | 1^o^ |  |  |  |  |  |  |  |  |  |  |  |  |  |  |  |  |  |  | 3 |
|  | **Total** | 172 | 8 | 10 | 31 | 2 | 5 | 1 | 6 | 7 | 6 | 2 | 3 | 2 | 1 | 1 | 4 | 1 | 1 | 1 | 1 | 1 | 1 | 1 | 18 | 13 | 6 | 305 |

Values in cells above are presented per patient (n=305). Rows represent ssRAB index biopsy result. Columns represents reference standard for each patient

**Table 1 – Diagnostic Contingency Table**

Diagnostic yield: ATS/ACCP Yield Numerator = Sum of values in rows: Malignant, ATS/ACCP Specific Benign. Intermediate yield numerator: Sum of values in rows: Malignant, ATS/ACCP Specific Benign and Diagnostic-Benign (intermediate)

Sensitivity for malignancy: True positive = sum of values in rows labeled “Malignant” and in columns labeled Malignant Reference Standard. False Negative = sum of values in rows labeled ATS/ACCP Specific Benign, ATS/ACCP non-diagnostic, non-diagnostic and under column labeled Malignant State

* Other: Benign hepatic cells (1), Lymphoid tissue with no sign of malignancy (1)

^a^ Six (6) lost to follow-up include the following index biopsies: One (1) Pyrenophora species/Marielliottia dematoididea, One (1) Necroinflammaory tissue/debris, One (1) suspected hamartoma (fibroadipose tissue), One (1) Chronic inflammation, One (1) presence of macrophages only, One (1) non-specific lung tissue. Cases lost to follow-up were considered a benign reference state in this analysis.

^b^ Mycobacteria Avium Complex

^c^ Tuberculosis

^d^ Mycobacteria Simulae

^e^ Suspected infection with response to treatment, however no specific diagnosis obtained

^f^ ssRAB biopsy showed epitheliod histiocytes and fungal pseudohyphae morphologically suggestive of Candida species.   GMS stain showed fungal pseudohyphae suggested of candida however forceps biopsy not performed therefore no tissue available to confirm tissue invasion. Benign specific diagnosis per ATS/ACCP not obtained.

^g^ Abundant polymorphous lymphocytes, lymphoid tissue with germinal cells suggestive of hyperplasia on index biopsy. Flow cytometry demonstrated lymphocytes and granulocytes and CD4:CD8 ratio = 7.5. Benign specific diagnosis per ATS/ACCP not obtained. Reference state determined by VATS; index biopsy pathology consistent with surgical pathology

^h^ Confirmed by VATS

^i^ During index biopsy, dark brown fluid drained from nodule however benign specific diagnosis per ATS/ACCP not obtained. Nodule resolved on follow-up CT and fiducial placed during index procedure visible at biopsy location on follow-up CT

^j^ Index (ssRAB) biopsy ruled definitively/specific benign by pathologist

^k^ Change to false negative between 1 and 2 year follow-up. Specific number of cases provided in parenthesis.

^l^ Reference standard confirmed through tissue biopsy

^m^ Index (ssRAB) forceps biopsy showed benign fibroelastotic scar as the biopsy result

^n^ Cryptogenic organizing pneumonia

^o^ Cancer was not confirmed however oncologic treatment for lung cancer was administered. Cases are considered to have a malignant reference state.
